# Histone modifications facilitate the coexpression of bidirectional promoters in rice

**DOI:** 10.1186/s12864-016-3125-0

**Published:** 2016-09-30

**Authors:** Yuan Fang, Lei Wang, Ximeng Wang, Qi You, Xiucai Pan, Jin Xiao, Xiu-e Wang, Yufeng Wu, Zhen Su, Wenli Zhang

**Affiliations:** 1 State Key Laboratory for Crop Genetics and Germplasm Enhancement, Nanjing Agriculture University, Nanjing, Jiangsu 210095 China; 2 State Key Laboratory of Plant Physiology and Biochemistry, CBS, China Agricultural University, Beijing, 100193 China; 3 JiangSu Collaborative Innovation Center for Modern Crop Production (JCIC-MCP), Nanjing Agriculture University, Nanjing, Jiangsu 210095 China

**Keywords:** Bidirectional promoters,regulation of gene expression, Coexpression, histone marks, Nucleosome positioning, *Oryza sativa*

## Abstract

**Background:**

Bidirectional gene pairs are highly abundant and mostly co-regulated in eukaryotic genomes. The structural features of bidirectional promoters (BDPs) have been well studied in yeast, humans and plants. However, the underlying mechanisms responsible for the coexpression of BDPs remain understudied, especially in plants.

**Results:**

Here, we characterized chromatin features associated with rice BDPs. Several unique chromatin features were present in rice BDPs but were missing from unidirectional promoters (UDPs), including overrepresented active histone marks, canonical nucleosomes and underrepresented H3K27me3. In particular, overrepresented active marks (H3K4ac, H4K12ac, H4K16ac, H3K4me2 and H3K36me3) were truly overrepresented in type I BDPs but not in the other two BDPs, based on a Kolmogorov-Smirnov test.

**Conclusions:**

Our analyses indicate that active marks (H3K4ac, H4K12ac, H4K16ac, H3K4me3, H3K9ac and H3K27ac) may coordinate with repressive marks (H3K27me3 and H3K9me1/3) to build a unique chromatin structure that favors the coregulation of bidirectional gene pairs. Thus, our findings help to enhance the understanding of unique epigenetic mechanisms that regulate bidirectional gene pairs and may improve the manipulation of gene pairs for crop bioengineering.

**Electronic supplementary material:**

The online version of this article (doi:10.1186/s12864-016-3125-0) contains supplementary material, which is available to authorized users.

## Background

Bidirectional promoters (BDPs) regulate the bidirectional transcription of protein-coding gene pairs with head-to-head orientation, which means that the transcription of each gene occurs on a different DNA strand and in opposite directions. These promoters have been well characterized in the eukaryotic genomes of yeast [1, 2], *Drosophila* [3], humans [4, 5] and some plants [6, 7]. Investigations of BDPs in yeast and humans have shown that BDPs possess unique features compared to unidirectional promoters (UDPs). The sequences of BDPs have higher GC contents and fewer TATA boxes than those of UDPs [4, 5, 8]. The presence of overrepresented motifs, such as GABPA and YY1, has already been recognized as a characteristic of human BDPs [9–11]. Compared to UDPs, human BDPs have more epigenetic marks and chromatin related features, including RNA PolII binding sites, acetylation at H3, H3K9 and H3K27 and methylation at H3K4me2/3 [9, 12]. By contrast, H4 acetylation is underrepresented in human BDPs [11]. The majority of bidirectional gene pair products function in the same cellular pathway, and their involvement has been implicated in diverse processes, including DNA repair, the cell cycle, housekeeping, various metabolic pathways and human diseases [4, 10, 13–19]. Although the coexpression of bidirectional gene pairs is common in eukaryotic genomes [5, 20–23], the detailed underlying mechanisms that regulate coexpression are not well characterized. Thus, uncovering the unique regulatory mechanisms associated with BDPs will provide new insights for understanding eukaryotic gene regulation, especially co-regulation.

Progress has been made in characterizing plant BDPs in *Arabidopsis* [6, 24, 25], rice [6], maize [7] and *Populus* [6] due to the recent availability of whole plant genome sequences and transcriptome data. Similar to BDPs in yeast and humans, plant BDPs have higher GC contents and fewer TATA boxes than UDPs [6, 20, 24, 26]. Moreover, plant BDPs are involved in the regulation of important agricultural traits [27–31]. However, information on the chromatin related features of plant BDPs is still lacking.

In this study, we continued to perform a comprehensive analysis of chromatin-based epigenetic features in rice BDPs. BDPs were classified into three types (I, II and III with sizes of 0–250 bp, 250–500 bp and 500–1000 bp, respectively) as described previously [32]. The BDP size was defined as the intergenic distance between the transcription start sites (TSSs) of the corresponding gene pairs. We observed that type I BDPs (BDPs I) showed the highest percentage and strongest level of coexpression, which was in agreement with the highest level of coexpression from gene pairs with 200 bp separating their TSSs. We also found several unique chromatin features present in rice BDPs that are not found in UDPs, including the overrepresentation of active histone marks, canonical nucleosomes and the underrepresentation of H3K27me3. Strikingly, we found that overrepresented H3K4ac,H4K12ac, H4K16ac, H3K9ac and H3K27ac marks may play a significant role in the regulation of coexpressed gene pairs, indicating that histone acetylation functions in the co-regulation of gene pairs. Thus, our findings help to enhance the understanding of a unique epigenetic mechanism used in the regulation of BDPs, which could be used to improve the manipulation of gene pairs in crop bioengineering.

## Results

### DNA sequence features of rice bidirectional promoters

To comprehensively characterize the DNA sequence profiles of the BDPs in rice, we first identified bidirectional gene pairs with head-to-head orientations using the updated version of the rice genome (The Institute for Genomic Research (TIGR), rice subsp.*Japonica* version 7.0) as described previously [32], which contains a total of 55,801 annotated genes. We identified a total of 290 type I BDPs, 294 type II BDPs (BDPs II) and 627 type III BDPs (BDPs III), with TSS intergenic distances of 0–250 bp (BDPs I), 250–500 bp (BDPs II) and 500–1000 bp (BDPs III), respectively. Our results were similar to the previously reported number of rice BDPs [24].

**Fig. 1:**
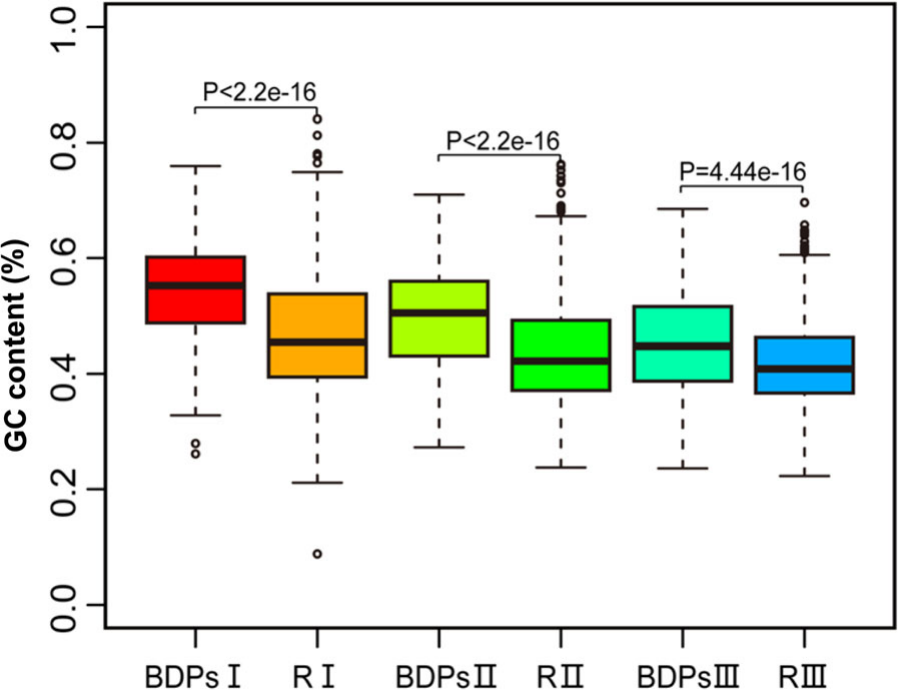
Comparison of GC contents between BDPs and randomly selected UDPs. Type I, II and III BDPs: bidirectional promoters with intergenic sizes ranging from 0 to 250 bp; from 250 to 500 bp and from 500 to 1000 bp, respectively. R I, RII and RIII: randomly selected unidirectional promoters (UDPs) with sizes as 250 bp, 500 bp and 1000 bp starting from upstream of TSS of the downstream genes, respectively, were used as controls for type I,II and III BDPs, respectively. Statistical analysis was conducted with a two-sample K-S test

We then calculated the GC contents and observed approximately 54 %, 50 % and 45 % GC contents in types I, II and III BDPs, respectively (Additional file 1: Table S1). The GC contents in BDPs was significantly higher than from randomly selected UDPs (Fig. 1).This result confirmed the presence of GC-enriched sequences in eukaryotic BDPs [5, 9, 24]. In addition, the TATA box content was analyzed using the PLACE database [33]; TATA boxes were found in approximately 18 %, 52 % and 82 % of type I, II and III BDPs, respectively (Additional file 1: Table S1). The ratio of genes containing TATA boxes in type I BDPs was 30 % less compared to randomly selected type I UDPs (random I) (Additional file 1: Table S1). In general, our analysis showed that GC content is inversely related to BDP size. By contrast, TATA content is positively associated with BDP size. Our results are the first to demonstrate that many rice type I BDPs are GC-rich sequences lacking TATA boxes. After comparing the expression of gene pairs among the three BDP types, we found that type I BDPs had the highest expression level; whereas type III BDPs had the lowest expression level. In addition, we observed that the expression of one of the gene pairs was significantly higher (higher FPKM, *p* < 0.01)than its counterpart (lower FPKM, *p* < 0.01) (data not shown). This result indicated that the GC or TATA content may affect the expression level of the corresponding genes.

### Overrepresented motifs in rice BDPs involve in stress responses

To determine the occurrence of conserved motifs within rice BDPs, which are potential binding sites for trans-factors involved in the regulation of bidirectional gene expression, we first classified BDPs into constitutive and tissue-specific categories according to the expression profiles of the bidirectional gene pairs in three rice tissues under normal conditions (leaf, callus and root) (Additional file 2: Table S2). We then identified the presence of overrepresented motifs with *p*-value cut-off of 0.05 using the PLACE and PlantCare databases [34]. When 1000 randomly selected UDPs were used as a control (Additional file 3: Table S3), we identified three overrepresented constitutive motifs (SORLIP2AT (GGGCC), SITEIIATCYTC (TGGGCY) and UP1ATMSD(GGCCCAWWW) in BDPs from the three rice tissues tested(Additional file 4: Table S4). This result was similar to previously reported findings [6]. These motifs are possibly involved in regulating phyA-responsive transcripts, the expression of PCNA (proliferating cell nuclear antigen) genes and the regulation of genes in auxiliary buds. In addition, we observed that TBF1HSF (GAAGAAGAA) was overrepresented in leaf tissue, whereas the ACGTABREMOTIFA2OSEM (ACGTGKC) and BOXIIPCCHS (ACGTGGC) motifs were dominant in callus tissue (Additional file 4: Table S4). The TBF1HSF motif (GAAGAAGAA) is associated with the expression of genes related to diverse defense responsive [35] and the regulation of thermo-tolerance in *Arabidopsis* [36]. The ACGTABREMOTIFA2OSEM motif (ACGTGKC) has been implicated in the regulation of genes associated with different metabolic pathways during drought stress in soybean [37], and the regulation of genes associated with ABA-responsive in *Arabidopsis* [38].

To investigate whether BDP-related gene pairs are involved in stress responses in rice, we analyzed differentially expressed bidirectional gene pairs under drought stress using publicly available RNA-seq data (GSE65022). When compared to control genes, 62 up-regulated gene pairs and 70 down-regulated gene pairs with fold change greater than 2 were identified under drought stress. We then identified 14 overrepresented motifs in the promoter regions of gene pairs that were both up-and down-regulated during drought stress (Additional file 5: Table S5). However, when compared with non-drought inducible BDPs (Additional file 3: Table S3), we found that 8 motifs (highlighted in red) were present in both drought-inducible and non-drought-inducible BDPs. Only the six remaining motifs were truly related to stress response, indicating that the gene pairs with promoters contain these motifs play diverse roles in plant development and stress responses. In addition, some of the well-characterized motifs involved in plant stress responses, such as ACGTABREMOTIFA2OSEM (ACGTGKC) [37], CACGTGMOTIF (CACGTG) [39], CAMTA1(CCGCGT) [40] and ABRERATCAL(MACGYGB) [41, 42] (Additional file 5: Table S5) were overrepresented in the promoters of both drought-inducible rice gene pairs and unidirectional genes that were upregulated under drought stress (Additional file 3: Table S3), This result was consistent with prior reports of overrepresented motifs in humans and plant BDPs compared to UDPs [5, 9, 24]. The presence of tissue-specific overrepresented motifs may play an important role in regulating plant development and stress-responses. The binding of various trans-factors to these motifs may be a unique mechanism responsible for the constitutive, tissue-specific and stress responsive expression of bidirectional genes.

### Coexpression of rice bidirectional gene pairs

**Fig. 2:**
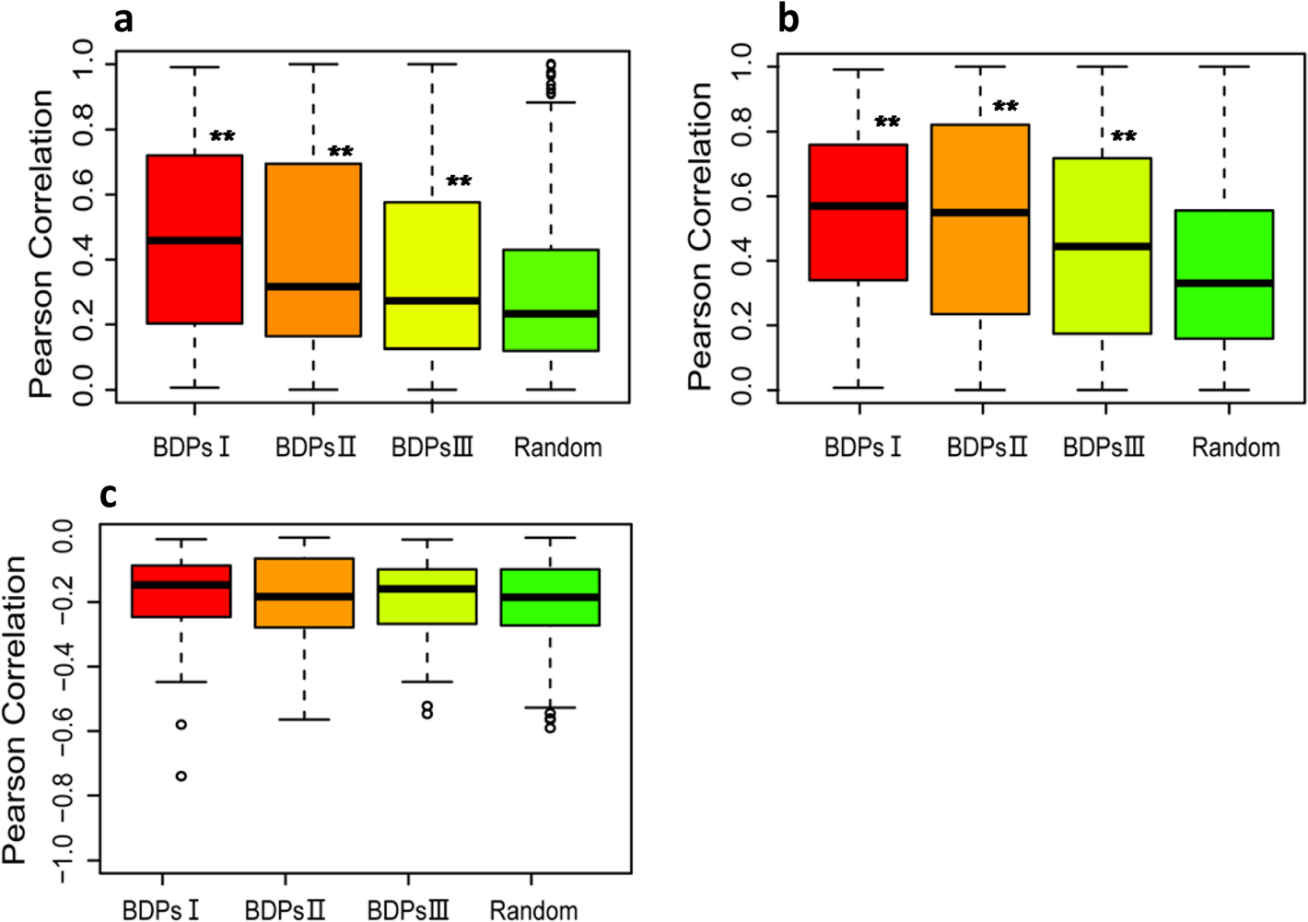
Coexpression analysis of bidirectional gene pairs. **a**. Comparison of expression correlation between gene pairs from type I, II and III BDPs, and randomly selected unidirectional genes. The Pearson correlation coefficients were calculated from all gene pairs using the absolute expression values. A statistical analysis was performed using a two-sample K-S test, where ** *p* < 0.001. **b.** Comparison of coexpression correlation between coexpressed gene pairs and randomly selected unidirectional genes. The expression mode of each gene pair was classified into two categories, coexpression or anti-expression, based on the Pearson correlation coefficients. A positive Pearson correlation coefficient indicated coexpression (Fig. 2**b**), and a negative Pearson correlation coefficient indicated anti-expression (Fig. 2**c**). All gene pairs with positive Pearson correlation coefficients were selected for analysis. Significant difference were determined using a two-sample K-S test, where ** *p* < 0.001. **c**. Comparisons of the expression correlations between anti-expressed gene pairs and randomly selected unidirectional genes. All gene pairs with negative Pearson correlation coefficients were selected for analysis. A statistical analysis was performed using a two-sample K-S test, where ** *p* < 0.001

Bidirectional gene pairs in animals and *Arabidopsis* are usually highly coexpressed [20, 43]. However, the effect of the intergenic distance between the TSSs of a gene pair on the coexpression of the corresponding genes was unclear. We calculated the Pearson correlation coefficients for all bidirectional gene pairs using eleven total gene expression datasets extracted from the Rice Genome Annotation Project (http://rice.plantbiology.msu.edu/expression.shtml) as described previously [32]. We observed that the median coexpression values of bidirectional gene pairs were significantly higher than those of randomly selected two adjacent unidirectional genes (Fig. 2a). This result suggested that bidirectional gene pairs driven by BDPs tend to be more coexpressed than randomly selected two adjacent unidirectional genes. Based on the strength of the correlation (Pearson correlation coefficient) between the expression levels of the gene pairs, we divided the expression mode for bidirectional gene pairs into four categories: coexpression, anti-expression, independent expression and no expression (Additional file 6: Table S6). The coexpression rather than anti-expression were significantly different between BDPs and UDPs (Fig. 2b and c). In addition, the percentage of coexpressed gene pairs decreased with increasing BDP size (Additional file 6: Table S6). The highest frequency of coexpression was previously found in gene pairs separated by 200 bp [32], here we further observed that gene pairs were generally more frequently coexpressed when the intergenic distance was less than 500 bp. (Additional file 7: Figure S1). The high frequency of coexpression from BDPs with a 200 bp intergenic distance between the TSSs of each gene may be explained by the 200 bp spacing of nucleosomes; a similar finding was previously reported in *Arabidopsis* [20]. By contrast, no significant difference was observed between BDPs with more than 700 bp of TSS intergenic space and UDPs. We speculated that 200 bp is probably the optimal space for sharing regulatory elements and recruiting transcriptional machinery to enhance the coexpression of bidirectional gene pairs.

Taken together, the above analyses indicated that bidirectional gene pairs, especially in type I BDPs, are highly coexpressed in rice. However, the underlying mechanisms need to be further investigated.

### Overrepresented histone marks associated with rice BDPs

Histone modifications play fundamental roles in controlling the chromatin-based regulation of gene expression in eukaryotic genomes. To profile the histone marks around BDPs, we performed chromatin immunoprecipitation (ChIP) followed by high through-put sequencing (ChIP-seq) for six histone marks as described previously [32], which included three active marks (H3K27ac, H3K4ac and H3K9ac) and three repressive marks (H3K9me1, H3K9me3 and H3K27me3). In addition, we also included six of active marks (H4K12ac, H3K4me3, H3K36me3, H3K4me2, H4K16ac and H3K23ac) previously characterized in rice [44, 45]. We selected rice unidirectional genes with expression levels (FPKM value) comparable as control. We observed that profiling of all marks was possible regardless of the number of control genes used because the distribution of each mark was similar when between one time (1×) and five times (5×) the number of bidirectional genes were analyzed (Additional file 8: Table S7). Thus, we decided to use 1X control genes for the following analysis.

**Fig. 3:**
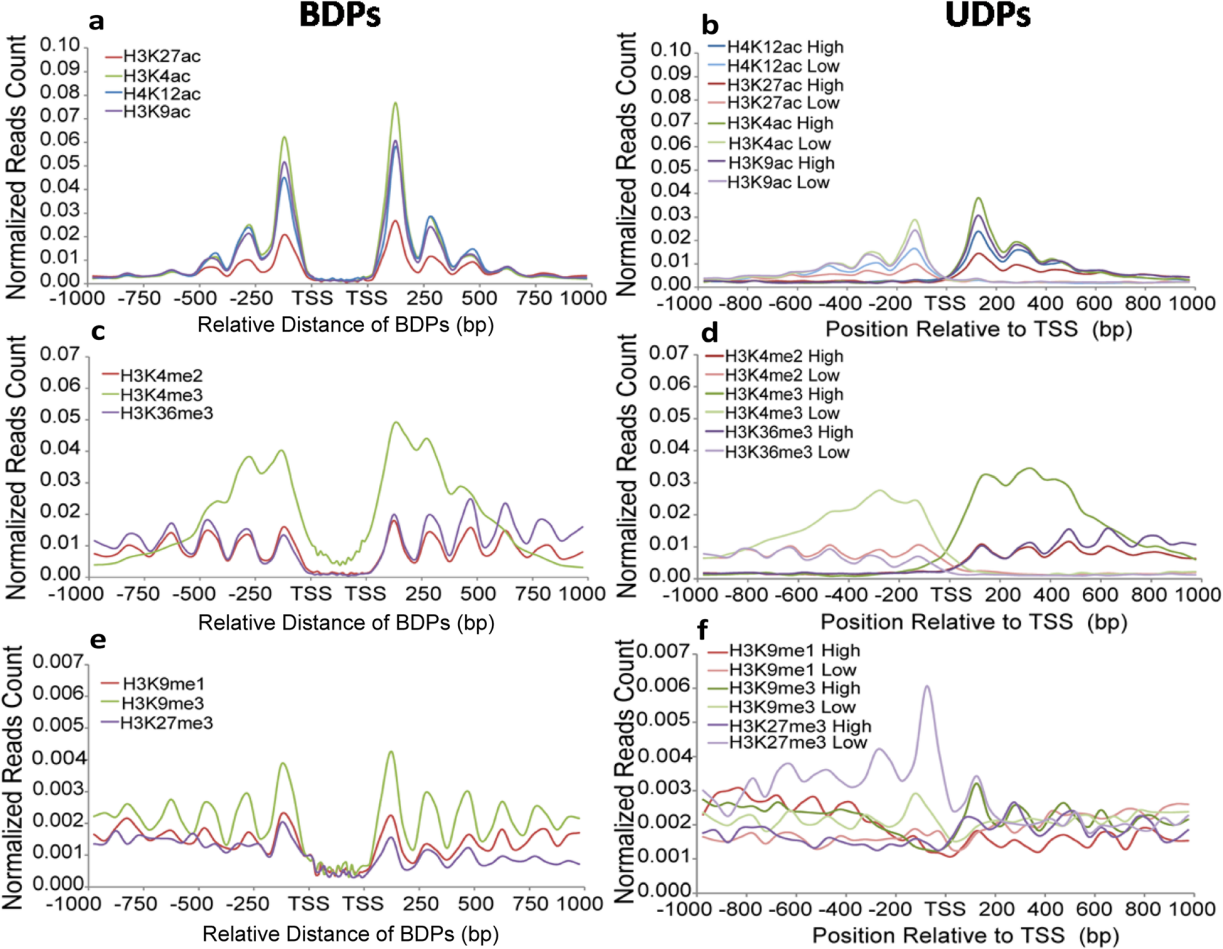
Profiling of histone marks across type I BDPs and UDP controls with the same gene number and expression level as the bidirectional gene pairs. Unidirectional genes with higher and lower FPKM values were aligned on the right and left side, respectively (Fig. 3 **b**, **d** and **f**). Bidirectional gene pairs with higher and lower FPKM values were aligned on the right and left sides of the BDPs, respectively (Fig. 3 **a**, **c** and **e**). Normalized reads counts indicated the enrichment of each mark were calculated by reads number per bp of genomic region per million reads. X-axes show the relative distances of BDPs (bp) in Fig. 3 **a**, **c** and **e** and their positions relative to the TSSs in Fig. 3 **b**, **d** and **f**; Y-axes show normalized reads counts (read number in per bp of genome per million reads) within 1 kb upstream and downstream of the TSS. **a**. Profiles of active marks: H4K12ac, H3K27ac, H3K4ac and H3K9ac in type I BDPs (Fig. 3 **a**) and UDPs (Fig. 3 **b**). **b**. Profiles of active marks: H3K4me2, H3K4me3 and H3K36me3 in type I BDPs (Fig. 3 **c**) and UDPs (Fig. 3 **d**). **c**. Profiles of repressive marks: H3K9me1, H3K9me3 and H3K27me3 in type I BDPs (Fig. 3 **e**) and UDPs (Fig. 3**f**)

To confirm the accuracy of the ChIP-seq analysis, a qPCR assay was performed following a ChIP experiment using antibodies against H3K27ac and H4K12ac. In general, we found that ChIP-qPCR enrichment (% of input) for an individual BDP locus was consistent with the ChIP-seq result for that locus (normalized reads counts) (Additional file 9: Table S8). We then plotted the normalized reads across bidirectional gene pairs. Strikingly, we observed that the peak levels of each active mark (acetylation at H4K12, H3K27, H3K4 and H3K9, methylation at H3K4 and H3K36) were higher in type I BDPs than in UDPs (Fig. 3a and c). A similar trend was observed for type II and type III BDPs compared to the corresponding UDPs (Additional file 10: Figure S2a and c; Additional file 11: Figure S3 a and c), but the marks were more enriched in the genes in type I BDPs than type II and III BDPs. This result, which demonstrated that active marks are more enriched in rice BDPs compared to UDPs, is similar to findings in humans [9, 12]. Although occupancy of repressive marks (methylation at H3K9 and H3K27) was 10 times less than that of active marks, the amplitude of the oscillating peaks for H3K27me3 in type I BDPs was lower than in UDPs; this finding is contrary to the H3K9me3 enrichment observed in type I BDPs as compared to UDPs (Fig. 3e). Similarly, when compared to UDPs, less H3K27me3 and more H3K9me1/3 were also observed in type II and III BDPs (Additional file 10: Figure S22e; Additional file 11: Figure S3e).

To confirm that all of histone marks analyzed were truly overrepresented in BDPs, we performed a K-S test on the normalized reads counts from all histone marks distributed in the gene bodies of BDPs and UDPs (Additional file 12: Table S9). We found that significant changes in occupancy were only detected for H4K12ac, H4K16ac, H3K4ac, H3K4me2, H3K36me3 and canonical nucleosomes. Intriguingly, histone marks were mainly overrepresented in type I BDPs rather than in the other two BDPs. The K-S test result demonstrated that these five marks are truly overrepresented in type I BDPs compared to UDPs and the other two types of BDPs. In summary, the above analyses demonstrated that BDPs have characteristic chromatin features, especially in histone modifications, which may build a unique chromatin structure that affects the transcription of gene pairs.

### Histone marks associated with coexpression of bidirectional gene pairs

**Fig. 4:**
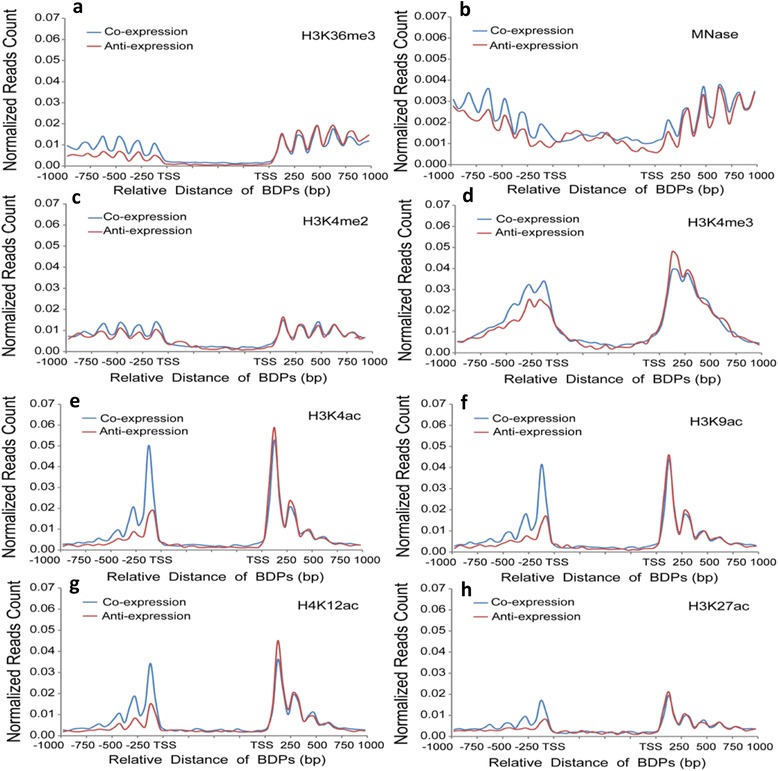
Profiling of histone marks and nucleosome occupancy between coexpressed and anti-expressed bidirectional gene pairs. Either coexpressed or anti-expressed bidirectional gene pairs with higher and lower FPKM values were aligned on the right and left sides of BDPs, respectively. **a**: H3K4me2; **b**: H3K4me3; **c**: H3K36me3; **d**: H3K4ac; **e**: H3K9ac; **f**: H4K12ac; **g**: H3K27ac; **h**: nucleosome occupancy. Normalized reads counts indicating the enrichment of each mark were calculated by reads number per bp of genomic region per million reads. X-axes show the relative distances from the BDP (bp); Y-axes show normalized reads counts (read number per bp of genome per million reads) within 1 kb upstream and downstream of the TSS

For all of the analyzed chromatin features distributed around BDPs (Additional file 8: Table S7 and Additional file 12: Table S9), we observed that the largest significant enrichment of histone marks in type I BDPs and not in the other two types, which is consistent with the presence of more coexpressed gene pairs in type I BDPs. We suspected that some of the marks were responsible for the coexpression of bidirectional gene pairs. To test this hypothesis, we profiled all active marks between coexpressed and anti-expressed gene pairs. Interestingly, we observed a similar histone mark profile between coexpressed and anti-expressed genes with higher FPKM values. The occupancy of marks, however, was higher in coexpressed genes with lower FPKM values compared to anti-expressed counterparts (Fig. 4). In addition, the K-S test on gene bodies indicated a significant difference in the occupancy of all marks between higher FPKM values and lower FPKM values of anti-expressed gene pairs (Additional file 13: Table S10), suggesting that the presence of those marks is closely associated with the level of gene expression. By contrast, only H3K4me2, H3K23ac, H3K36me3 and nucleosome occupancy were significantly different between co-expressed gene pairs with higher FPKM values and lower FPKM values (Additional file 13: Table S10). However, there was no significant difference in other marks, including six active marks (H3K4ac, H4K12ac, H4K16ac, H3K9c, H3K4me3 and H3K27ac) and three repressive marks (H3K27me3, H3K9me1 and H3K9me3) observed between coexpressed gene pairs with higher FPKM values and lower FPKM values. In addition, we also performed the significant test for association of 12 histone marks plus nucleosome occupancy with coexpression in type I BDPs (data not shown), 12 of them (except for the H3K36me3 mark) were related to coexpression of type I BDPs, indicating that the correlation level between histone marks and co-expression was higher in type I BDPs compared to the whole BDPs tested. This is consistent with the highest percentage of coexpressed gene pairs (50 %) detected in type I BDPs. In contrast to unidirectional genes and anti-expressed gene pairs, this analysis demonstrated that six active and three repressive histone marks in coexpressed gene pairs were not related to gene expression, indicating that these nine marks may coordinate to create unique chromatin features responsible for the coexpression of bidirectional gene pairs. Thus, the significant test result showed that some of marks are associated with gene expression level, whereas others are possibly responsible for the coexpression of gene pairs.

### Nucleosome positioning and occupancy associated with BDPs

**Fig. 5:**
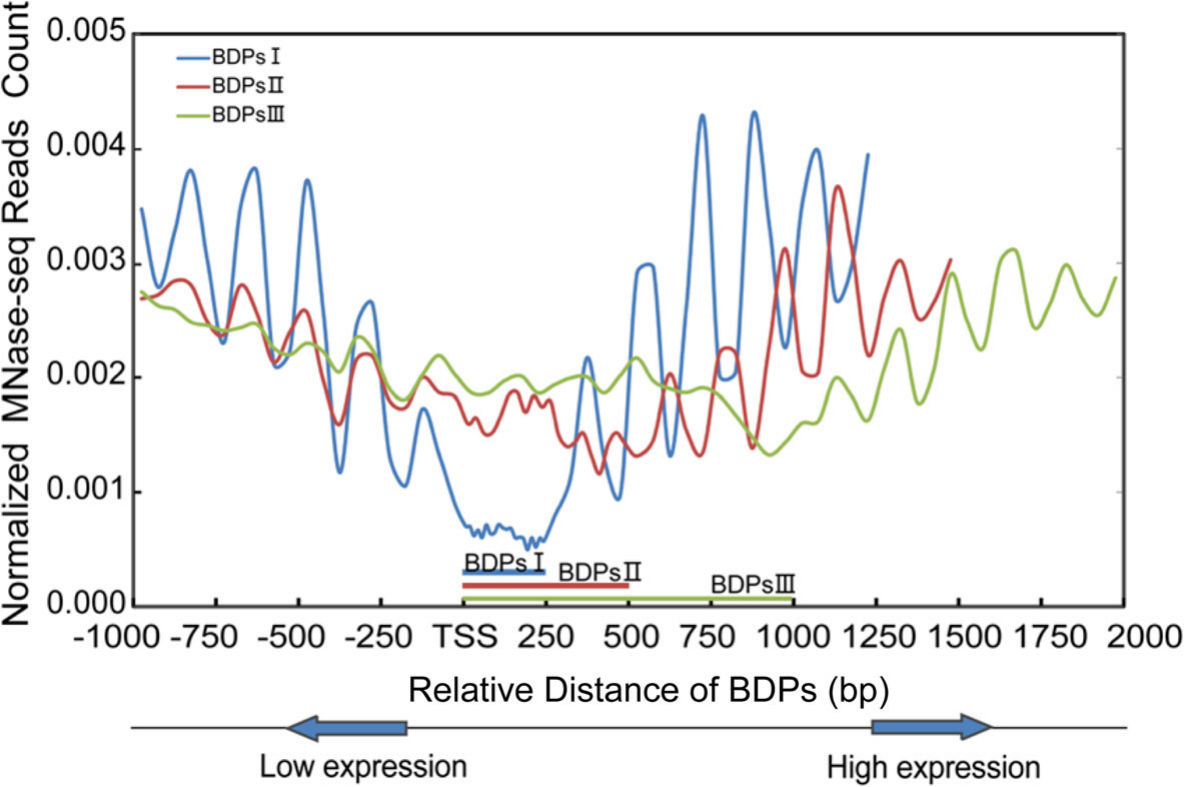
Profile of nucleosome positioning around each type of BDP. Profile of nucleosome positioning shown around type I BDPs (*blue line*),type II (*red line*) and type III (*green line*) BDPs extending ±1 kb from each BDPs. Bidirectional gene pairs with higher and lower FPKM values are aligned on the right and left side of BDPs, respectively. Normalized MNase-seq reads count representing the nucleosome positions were calculated by reads number per bp of genomic region per million reads. X-axes show the relative distances from the BDPs (bp); Y-axes show normalized MNase-seq reads counts (read number per bp of genome per million reads) within ±1 kb of the TSS. Paired-end MNase-seq reads were normalized and used for nucleosome positioning profiling. The bottom diagram indicates the direction of different expression levels from each gene pair: the gene with higher expression (higher FPKM values) is located on the right side and the gene with lower expression (lower FPKM values) is located on the left side

Nucleosome positioning and occupancy can modulate the regulation of gene expression in eukaryotes by either favoring or dis-favoring the accessibility of the underlying DNA elements to trans-factors [46, 47]. To examine the nucleosome positioning around BDPs, we performed a similar analysis as histone marks for profiling nucleosome positioning across bidirectional gene pairs. As expected, the nucleosome profiling exhibited a prominently less occupancy in each kind of BDPs than the flanking nucleosomal regions. Each BDP was immediately flanked by an array of regularly spaced, well-positioned nucleosomes with progressively elevated phasing status from the TSS to the gene body (Fig. 5). A similar trend was observed in unidirectional genes, but nucleosome occupancy around BDPs was significantly higher compared to UDPs (Fig. 5; Additional file 14: Figure S4). By calculating the highest amplitudes of the phased nucleosome (Additional file 8: Table S7), we found that the nucleosome occupancy increased by approximately 33 % and 27 % for genes in type I BDPs with higher FPKM and lower FPKM values, respectively, as compared to randomly selected unidirectional genes. A certain change was also observed in type II and III BDP genes with higher FPKM values, but no change in genes with lower-FPKM values (Additional file 8: Table S7). However, the K-S test indicated that the change in nucleosomal occupancy between bidirectional gene pairs and unidirectional genes was only significant for type I BDPs (Additional file 12: Table S9).

In summary, the nucleosome positioning status around the BDPs is similar to those flanking UDPs in animals and other plants [48–50]. A distinct nucleosome positioning symmetrically flanks around type I BDPs compared to the other two types of BDPs, which is possibly associated with the coexpression of BDP genes.

## Discussion

Growing evidence has demonstrated that genes with the similar expression level tend to be physically close to each other, and are typically coexpressed within various eukaryotic genomes, including yeast [51], humans [52, 53], *Drosophila* [54, 55], nematode [56, 57], mouse [58] and *Arabidopsis* [21, 59, 60]. However, the underlying mechanisms responsible for the coexpression of gene pairs have remained unclear. Thus, uncovering regulatory mechanisms associated with BDPs will provide new insights into the understanding of eukaryotic gene regulations, especially in the expression of gene pairs.

### Chromatin structures and bidirectional transcription of BDPs

Divergent transcription has been considered as an intrinsic property of many promoters in yeast and mammals [1, 2, 61–64]. Accumulating evidence from mammals indicates that divergent transcription is a consequence of genetically and epigenetically combined actions, mainly including inherent promoter DNA sequences and chromatin related changes [64–67]. BDPs are a good system for elucidating the chromatin-based regulatory mechanisms that control the bidirectional transcription of gene pairs in eukaryotes. Human BDPs contain unique features that UDPs lack, including overrepresented DNA motifs [9, 10], overrepresented active histone marks [9], and differences in the distribution of histone marks and functions of CTCF and cohesins between BDPs and UDPs [12]. However, the underlying chromatin based mechanisms for BDPs, especially in plants, were not well studied.

By integrating ChIP-seq and MNase-seq datasets, we provided the first comprehensive characterization of chromatin features in rice BDPs. Our results demonstrate that rice BDPs have typical chromatin features associated with active promoters: a low occupancy of canonical nucleosomes in BDPs, well positioned +/−1 canonical nucleosomes and the enrichment of active histone marks. This finding indicated that some of these marks may be involved in bidirectional initiation and elongation. In human divergent promoters which are flanked by coding genes and non-coding DNA sequences, no difference was observed in the distribution of transcription initiation related active marks (acetylation at H3/H4) and +/−1 nucleosome positioning between upstream non-coding sequences and downstream coding gene. However, elongation-related marks (H3K79me2, H3K36me3 and H2Bub) were only present in downstream gene bodies rather than the upstream non-coding sequences, suggesting that transcription elongation is a key determinant of the final fate of transcriptional direction from divergent promoters [68–70].

### Chromatin structure and coexpression of bidirectional gene pairs

Nucleosome-free or low occupancy of nucleosomes have been reported in the plant unidirectional promoter regions. Majority of plant promoter regions are associated with DNase I hypersensitive sites (DHSs), which are sensitive to cleavage by DNase I or other nucleases [44, 71–73]. Anti-correlation between nucleosome occupancy within promoters and gene expression [74], but correlation between nucleosomes density around TSS/ gene bodies and gene expression, was detected in plant unidirectional promoters [72, 75]. Similarly, BDPs displayed a similar nucleosome positioning pattern within promoter regions, and a similar relationship between nucleosome/active marks distribution and bidirectional gene expression. In addition, nucleosomes occupancy distributed within or out of UDP/ BDPs are directly associated with the level of active histone marks in the corresponding region. Thus, UDPs and BDPs generally share similar chromatin structural features in regulating the corresponding gene expression. However, it is unclear about the relationship between chromatin structural features and coexpression of plant bidirectional gene pairs.

A possible correlation between nucleosome occupancy and coexpression rates was observed in humans and yeast [76–78]. Additionally, the possible role of chromatin modifications in coexpression was explored in *Drosophila* [79], humans [53, 80–82] and yeast [83, 84]. However, direct evidence of the effects of histone marks on coexpression, especially in plants, was still missing.

Compared to UDPs, we observed that all active marks tested and nucleosome occupancy were overrepresented in BDPs (Additional file 8: Table S7), but a K-S test on gene body indicated that only a subset of these active marks (H4K12ac, H4K16ac, H3K4ac, H3K4me2, H3K36me3 and nucleosome occupancy) were significantly overrepresented in type I BDPs (Additional file 12: Table S9). This differs from human BDPs, in which H4 acetylation is underrepresented [11]. Strikingly, the true overrepresentation of active marks and canonical nucleosomes strongly correlated with the highest coexpression level of bidirectional gene pairs in type I BDPs. This result suggested that these overrepresented chromatin features may create chromatin structures that favor the coregulation of gene pairs. This prediction was further supported by the lack of significant changes in active marks (H3K4ac, H4K12ac, H4K16ac, H3K9ac, H3K4me3 and H3K27ac) observed in coexpressed genes, whereas all marks tested were significantly different in anti-expressed genes (Additional file 13: Table S10). Especially, consistent with the highest percentage of coexpressed gene pairs in type I BDPs, we observed a stronger correlation between histone marks and coexpression in type I BDPs compared with the whole BDPs tested. Among the 13 marks tested, only H3K36me3 was not related to coexpression in type I BDPs (data not shown). Usually, active marks are directly correlated with gene expression, whereas repressive marks are anti-correlated with expression in eukaryotes [85, 86]; this is not the case for the coexpression of gene pairs. Similarly, repressive marks do not display anti-correlated with gene expression in coepxressed gene pairs as compared with unidirectional genes and antiexpressed gene pairs. Our analyses demonstrated that overrepresented active marks (H3K4ac, H4K12ac, H4K16ac, H3K9ac, H3K4me3 and H3K27ac) may coordinate with repressive marks (H3K27me3 and H3K9me1/3) to build a unique chromatin features favorable for coregulation of bidirectional gene pairs. A similar histone modification-based mechanism, involving coacetylation or deacetylation was found to affect the coexpression of neighboring genes in yeast [87, 88].

## Conclusions

By integrating RNA-seq, ChIP-seq and MNase-seq datasets, we identified several unique chromatin features present in rice BDPs that are absent in UDPs, including overrepresented active histone marks, canonical nucleosomes and underrepresented H3K27me3. In particular, overrepresented acetylation at H3K4/K9/K27 and H4K12/K6 may play a significant role in regulating coexpression of gene pairs. Thus, our analyses indicated that the coexpression of bidirectional gene pairs is a consequence of the combined actions of multi-layer regulations, from DNA itself to specialized chromatin structures including nucleosome positioning and the coordination of active and repressive histone modifications.

## Methods

### Plant materials

Germinated rice cultivar “Nipponbare” seeds were sowed in soil and grown in a greenhouse for two weeks. Rice seedlings were then collected for ChIP-seq or ChIP-qPCR experiments as described below.

### Identification of bidirectional promoters

We retrieved the rice (*Oryza sativa*, subsp. *japonica*) genomic sequence and annotation data sets from the Rice Genome Annotation Database at TIGR (http://www.tigr.org/tdb/e2k1/osa1) as described previously [32]. Bidirectional genes were defined as gene pairs with head-to-head orientation, less than 1000 bp between their TSSs, and transcription from opposite strands. Bidirectional promoters (BDPs) were classified by the physical length of intergenic region between the TSSs of each gene pair into either type I (0–250 bp), II (250–500 bp) or III (500–1000 bp). All gene pairs annotated as protein coding genes were included for further analysis. Unidirectional promoters (UDPs) randomly selected from unidirectional genes with expression level similar to the bidirectional gene pairs were used as controls.

### Data analysis

#### RNA-seq

We downloaded publicly available RNA-seq datasets (GSM655033) from seedlings [44], that were grown in the same condition as those used for the ChIP-seq experiment. The drought-regulated expression of bidirectional gene pairs was analyzed using public RNA-seq data sets (GSE65022). The expression values (FPKM) of bidirectional gene pairs were calculated as described previously [44].

#### ChIP-seq, ChIP-qPCR and MNase-seq

ChIP-seq datasets (GSE79033) [32] for H3K4ac (Millipore, 07–539), H3K9ac (Millipore, 07–352), H3K27ac (Abcam,ab4729), H3K27me3 (Millipore, 07–449), H3K9me1 (Millipore, 07–395) and H3K9me3 (Millipore, 07–442), were generated from seeding using a previously described method [44]. Six of previously characterized ChIP-seq and MNase-seq (SRP045236)^46^ datasets obtained from seedlings for H3K4me3, H3K4me2, H3K36me3 and K4K12ac (GSE26734) [44] and H4K16ac and H3K23ac (GSE69426) [45] were downloaded from NCBI for further analysis. All ChIP-seq datasets were analyzed using the same pipeline as previously described [44].

To confirm the ChIP-seq results, we conducted a ChIP-qPCR assay following ChIP experiments using two histone marks (H3K27ac and H4K12ac). Five of the BDPs were randomly selected to design primers (Additional file 15: Table S11) for ChIP-qPCR analysis. One primer set was triplicated in the qPCR assay.

To profile the chromatin features of histone marks and nucleosome positioning associated with bidirectional gene pairs, we plotted the normalized ChIP-seq and MNase-seq reads across all bidirectional gene pairs and randomly selected unidirectional genes as controls.

### Coexpression analysis

To calculate the expression mode of gene pairs, we used eleven of the raw expression datasets deposited in NCBI from the Rice Genome Annotation Project (http://rice.plantbiology.msu.edu/expression.shtml) as described previously [32]. Tophat was used to map the sequencing reads to the version 7 pseudo-molecules of the rice genome [89]. Cufflinks was used to calculate the expression abundances for RNA-seq libraries [90]. The presence or absence of expression values were assigned for digital gene expression (DGE) libraries. Genes were called as ‘expressed’ if at least one sequencing reads was mapped uniquely within an exon. For Pearson correlations, the FPKM values of bidirectional gene pairs were used in a matrix analysis. Genes with FPKM = 0 across all libraries were excluded from the analysis. A customized Perl script was used to calculate the PCCs (Pearson correlation coefficients) for each bidirectional gene pairs. To categorize the expression modes of bidirectional gene pairs (Additional file 6: Table S6), we randomly selected 1000 adjacent unidirectional genes regardless of the transcriptional direction of each gene as controls to calculate the Pearson correlation coefficient. Any bidirectional gene pairs with a Pearson correlation coefficient greater than the average number of all positive values (0.38) were defined as coexpressed; Any bidirectional gene pairs with a Pearson correlation coefficient less than the average number of all negative values (−0.20) were defined as antiexpressed; Bidirectional gene pairs with a Pearson correlation coefficient between 0.38 and −0.20 were defined as independent; Bidirectional gene pairs without test of Pearson correlation coefficient were defined as Null.

### Motif discovery and overrepresentation analysis

A statistic algorithm based on Z score and *p*-value filtering [91, 92] was used to test the significance of identified cis-elements, and to discover elements involved in a certain gene set. In this study, 930 total plant motifs (cis-regulatory elements) with functional annotations were collected from several groups, including Plant Cis-acting Regulatory DNA Elements (PLACE) database [33], AthaMap webserver [93], PlantCARE database [34] and text-mining results. The same number and the same length of input sequences from rice UDPs promoters were randomly selected for 1000 times as negative controls for calculating the overrepresented motifs with Z-scores. According to inquiry sequence length, the background from the analyzed promoter regions was classified into three groups as performed for BDPs: 0–250 bp, 250–500 bp and 500–1000 bp. For example, when a list of inquiry sequences had length under 250 bp, cis-elements were scanned in the inquiry sequences, and the 250 bp promoter regions of rice genes and Z scores were calculated according to the following method.

$$ Z=\frac{\overline{X}-\mu }{\sigma /\sqrt{n}} $$

$\overline{X}$, mean value of a motif present in the list of inquiry sequences;

μ, mean value of the same motif present in 1000 random lists of rice gene promoter regions with the same length (250 bp, 500 bp, 1000 bp);

σ, standard deviation of the mean value for 1000 by randomly selected sequences;

n, count of inquiry sequences.

A list of inquiry sequence lengths within 500 or 1000 bp was analyzed as for 250 bp sequences above. Ultimately, motifs with a *p*-value of less than 0.05 were considered significantly enriched in the inquiry sequences compared to gene promoters in the whole rice genome.

### Normalized reads count

To normalize the reads counts distributed in BDP/UDP regions for assessing nucleosome positioning (MNased reads) and histone marks, we first identified all uniquely mapped reads in the region 1000 bp downstream of the TSSs. This region was equally divided into 20 sliding windows. We then calculated the number of reads within a specific sliding window divided by the length of the sliding window (bp), followed by the number of reads within the mapped genome (Mb). The sum of each BDP/UDP per sliding window was divided into the number of BDPs/UDPs. For all mapped reads, their positions in the rice genome were used to determine the midpoint of the reads.

### Significance test

A two-sample test was performed to test for significant differences in gene expression and chromatin features (histone modifications and nucleosome occupancy) between BDPs and UDPs as described previously [32]. Briefly, we calculated the normalized reads counts of each bidirectional gene pair and UDP controls distributed either across the whole gene body or within the highest peak ranging from 100 bp to 150 bp downstream of TSS, respectively. Normalized reads counts were derived by calculating the number of reads within the mapped genome (Million), and dividing by the length of the gene (bp). R was used for all two-sample Kolmogorov-Smirnov (K-S) tests within groups, and “two.sided” was selected as the alternative hypothesis. Two samples were considered significantly different if the two-tailed *p*-value was less than 0.05.

## Additional files

Additional file 1: Table S1. Contents of GC and TATA within rice BDPs. (PDF 403 kb) Additional file 2: Table S2. Summary of constitutive and tissue-specific BDPs used for motif identification. (PDF 522 kb) Additional file 3: Table S3. Summary of motifs identified in randomly selected 1000 UDPs, non-drought inducible BDPs and drought inducible UDPs. (PDF 514 kb) Additional file 4: Table S4. High frequency of overrepresented motifs within constitutive and tissue-specific BDPs. (PDF 334 kb) Additional file 5: Table S5. Overrepresented motifs in BDPs response to drought stress (PDF 348 kb) Additional file 6: Table S6. Expression modes of bidirectional gene pairs associated with rice BDPs. (XLS 307 kb) Additional file 7: Figure S1. Coexpression analysis of gene pairs associated with BDPs with size separated by every 100 bp intergenic length The Pearson correlation coefficient was calculated from all gene pairs corresponding to BDPs separated every 100 bp using the absolute expression value. Statistical analysis was provided by a two-sample K-S test, where ** *p <* 0.001, * *p <* 0.05. (XLS 110 kb) Additional file 8: Table S7. Fold difference of intensity of histone marks and nucleosome occupancy between bidirectional genes and unidirectional genes. (PDF 246 kb) Additional file 9: Table S8. Comparison between ChIP-seq and ChIP-qPCR assay in an individual BDP locus. (PDF 247 kb) Additional file 10: Figure S2. Profiling of histone marks across type II BDPs and UDP controls with the same gene number and same expression level as bidirectional gene pairs Unidirectional genes with higher and lower FPKM values were aligned on the right and left side, respectively (Additional file 10: Figure S2b, d and f). And bidirectional gene pairs with higher and lower FPKM values were aligned on the right and left sides of BDPs, respectively (Additional file 10: Figure S2a, c and e). Normalized reads counts indicated the enrichment of each mark were calculated by reads number per bp of genomic region per million reads. X-axes show the relative distance of BDPs (bp) in Additional file 10: Figure S2a, c and e and the position relative to TSS in Additional file 10: Figure S2b, d and f; Y-axes show normalized reads counts (read number in per bp genome in per million reads) within 1 kb upstream and downstream of TSS. A. Profiles of active marks: H4K12ac, H3K27ac, H3K4ac and H3K9ac in type II BDPs (Additional file 10: Figure S2a) and UDPs (Additional file 10: Figure S2b), respectively. B. Profiles of active marks: H3K4me2, H3K4me3 and H3K36me3 in type II BDPs (Additional file 10: Figure S2c) and UDPs (Additional file 10: Figure S2d), respectively. C. Profiles of repressive marks: H3K9me1, H3K9me3 and H3K27me3 in type II BDPs (Additional file 10: Figure S2e) and UDPs (Additional file 10: Figure S2f), respectively. (PDF 271 kb) Additional file 11: Figure S3. Profiling of histone marks across type III BDPs and UDP controls with the same gene number and same expression level as bidirectional gene pairs Unidirectional genes with higher and lower FPKM values were aligned on the right and left side, respectively (Additional file 11: Figure S3b, d and f). Bidirectional gene pairs with higher and lower FPKM values were aligned on the right and left sides of BDPs, respectively (Additional file 11: Figure S3a, c and e). Normalized reads counts indicated the enrichment of each mark were calculated by reads number per bp of genomic region per million reads. X-axes show the relative distance of BDPs (bp) in Additional file 11: Figure S3a, a and e and the position relative to TSS in Additional file 11: Figure S3b, d and f; Y-axes show normalized reads counts (read number in per bp of genome per million reads) within 1 kb upstream and downstream of TSS. A. Profiles of active marks: H4K12ac, H3K27ac, H3K4ac and H3K9ac in type III BDPs (Additional file 11: Figure S3a) and UDPs (Additional file 11: Figure S3b), respectively. B. Profiles of active marks: H3K4me2, H3K4me3 and H3K36me3 in type III BDPs (Additional file 11: Figure S3c) and UDPs (Additional file 11: Figure S3d), respectively. C. Profiles of repressive marks: H3K9me1, H3K9me3 and H3K27me3 in type III BDPs (Additional file 11: Figure S3e) and UDPs (Additional file 11: Figure S3f), respectively. (PDF 313 kb) Additional file 12: Table S9. Kolmogorov-Smirnov test of eu-/hetero-chromatin marks and nucleosome occupancy reads between gene body of bidirectional gene pairs and control genes. (PDF 272 kb) Additional file 13: Table S10. Kolmogorov-Smirnov test of eu-/hetero-chromatin marks and nucleosome occupancy between gene body of coexpression and anti-expression bidirectional gene pairs. (PDF 325 kb) Additional file 14: Figure S4. Comparison of profiling of nucleosome positioning between BDPs and UDPs. Unidirectional gene controls or bidirectional gene pairs with higher and lower FPKM values were aligned on the right and left sides of BDPs, respectively. Normalized MNase-seq reads count representing nucleosome positioning were calculated by reads number per bp of genomic region per million reads. X-axes in Additional file 14: Figure S4a, b and c show relative distance of BDPs (bp); Y axes in Additional file 14: Figure S4a, b and c show normalized MNase-seq reads counts. The number and expression level of unidirectional genes analyzed were the same as the corresponding bidirectional gene pairs. A. Profile of MNase-seq reads around type I BDPs and corresponding UDPs control with higher or lower FPKM values, respectively. B. Profile of MNase-seq reads around type II BDPs and corresponding UDPs control with higher or lower FPKM values, respectively. C. Profile of MNase-seq reads around type III BDPs and corresponding UDPs control with higher or lower FPKM values, respectively. (PDF 324 kb) Additional file 15: Table S11. Primer information used for the ChIP-qPCR assay. (PDF 246 kb)
