# Supplementary figures and images for: Histone modifications facilitate the coexpression of bidirectional promoters in rice
Source: BMC Genomics. 2016 Sep 30;17:768. doi: 10.1186/s12864-016-3125-0 (PMC5045660; doi:10.1186/s12864-016-3125-0)

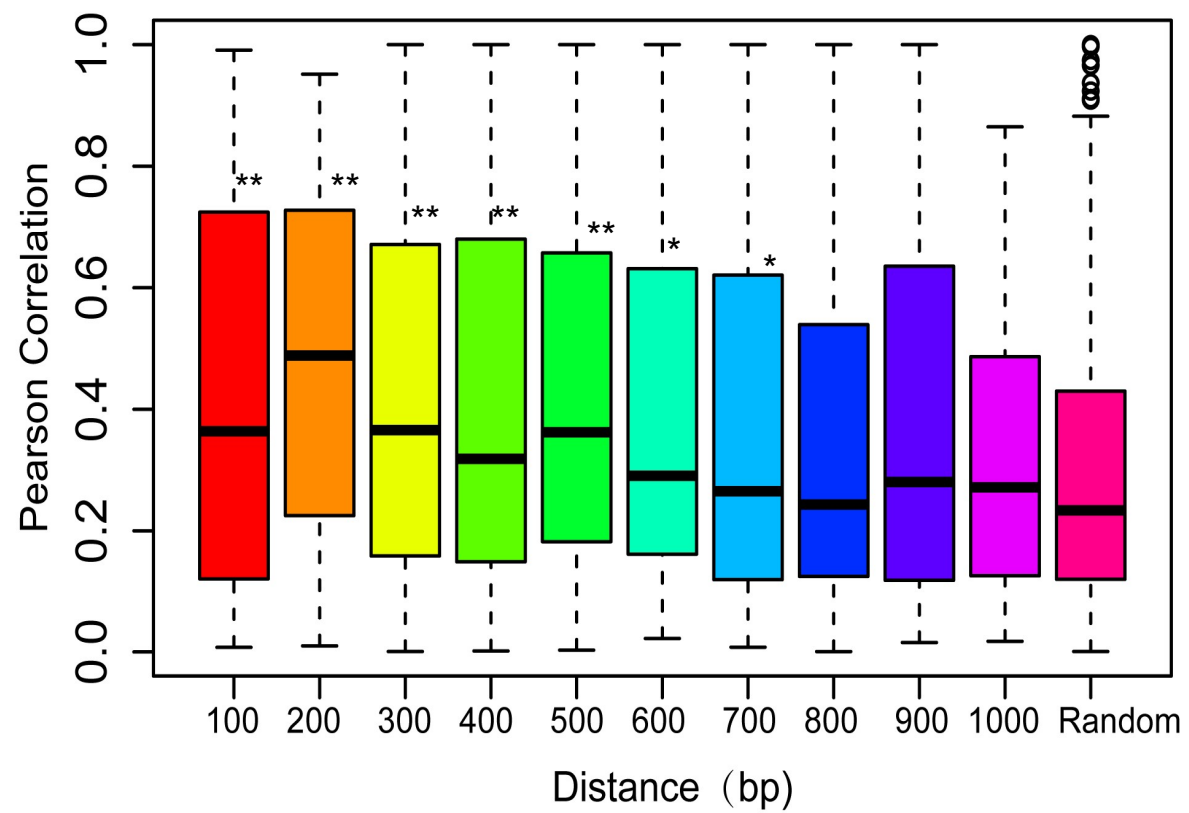

**Additional file 1: Figure S1.**

Supplement: Additional file 1: Table S1. — Contents of GC and TATA within rice BDPs. (PDF 403 kb) [file 12864_2016_3125_MOESM1_ESM.pdf]

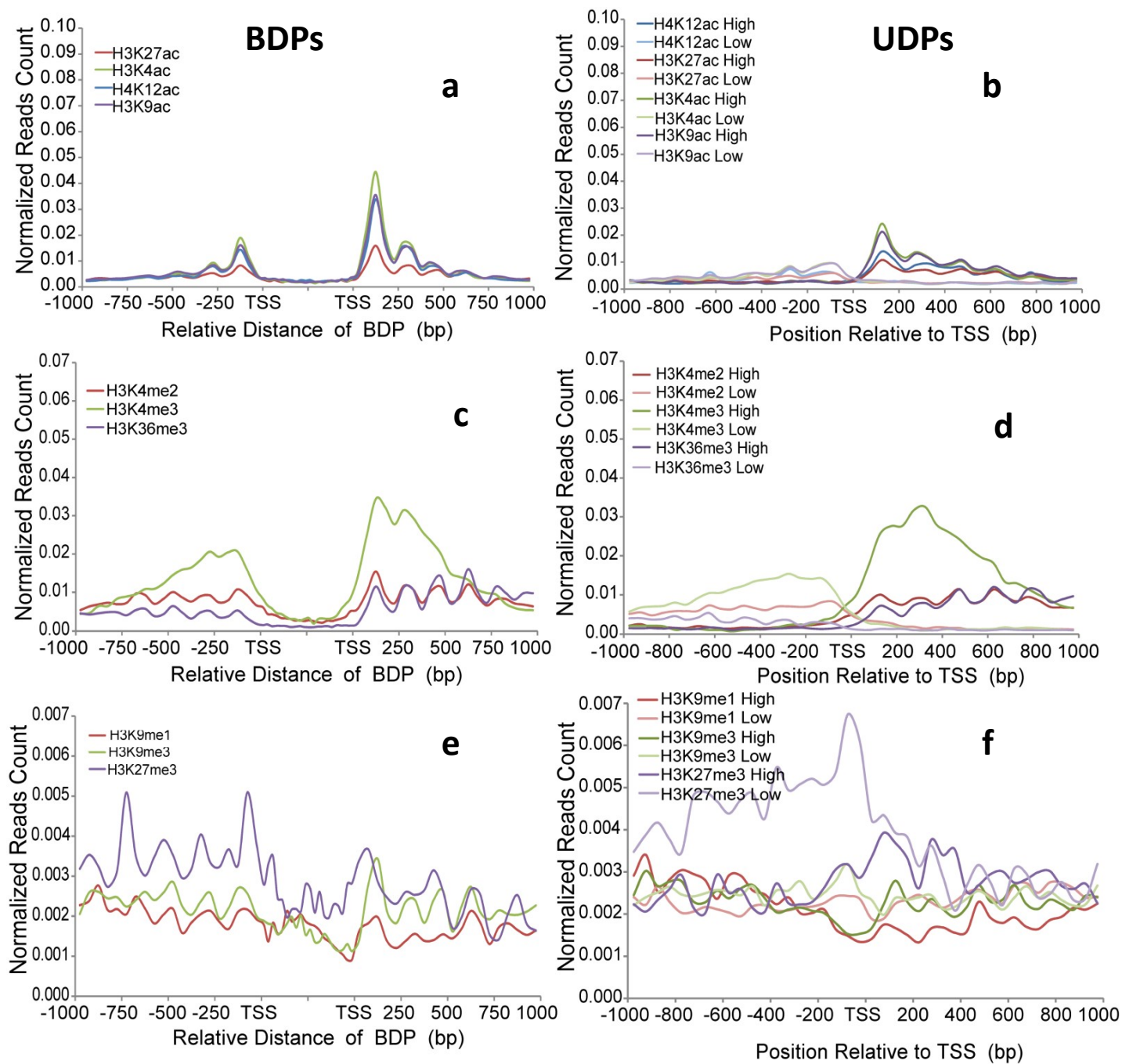

**Additional file 2: Figure S2.**

Supplement: Additional file 2: Table S2. — Summary of constitutive and tissue-specific BDPs used for motif identification. (PDF 522 kb) [file 12864_2016_3125_MOESM2_ESM.pdf]

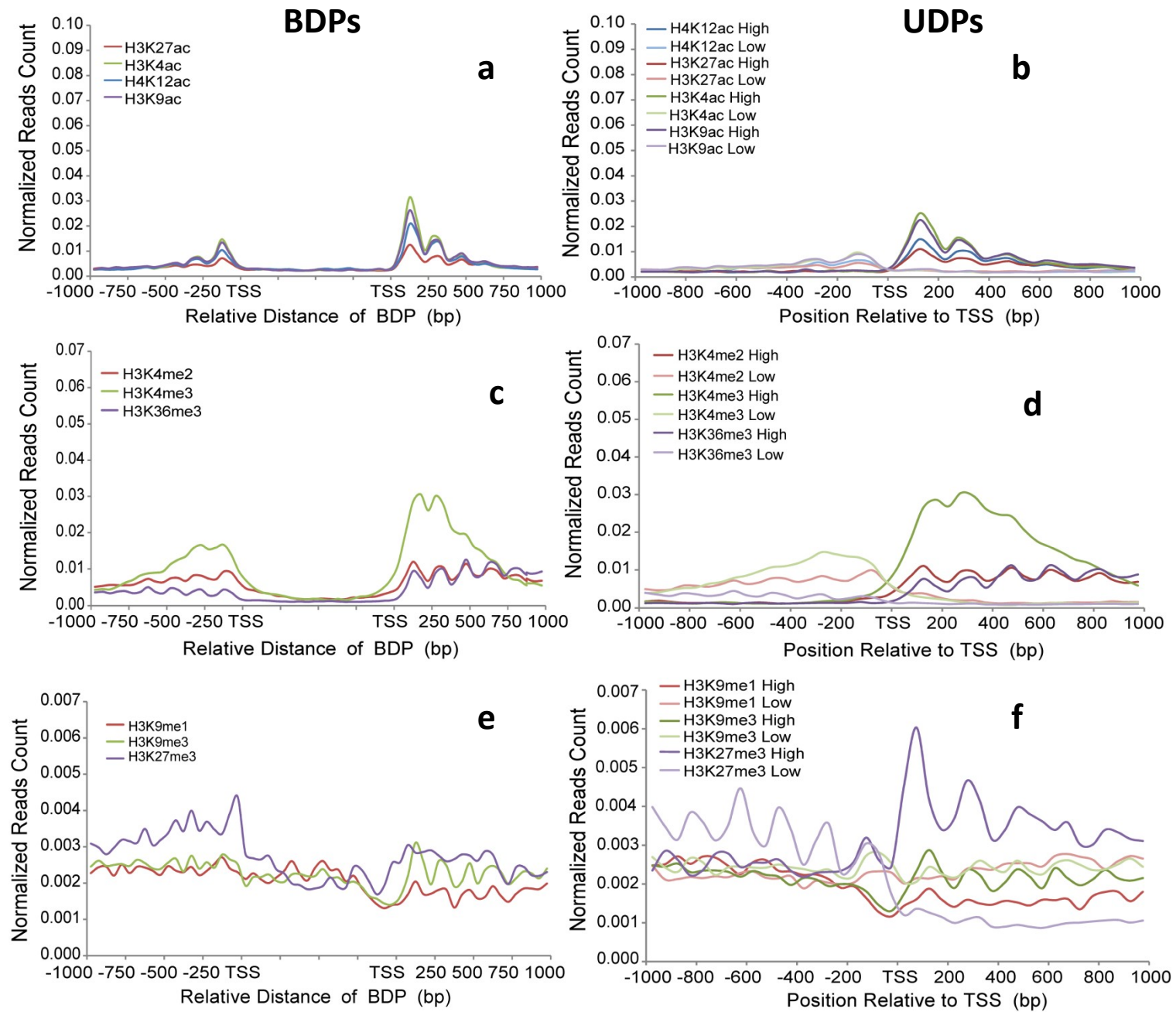

**Additional file 3: Figure S3.**

Supplement: Additional file 3: Table S3. — Summary of motifs identified in randomly selected 1000 UDPs, non-drought inducible BDPs and drought inducible UDPs. (PDF 514 kb) [file 12864_2016_3125_MOESM3_ESM.pdf]

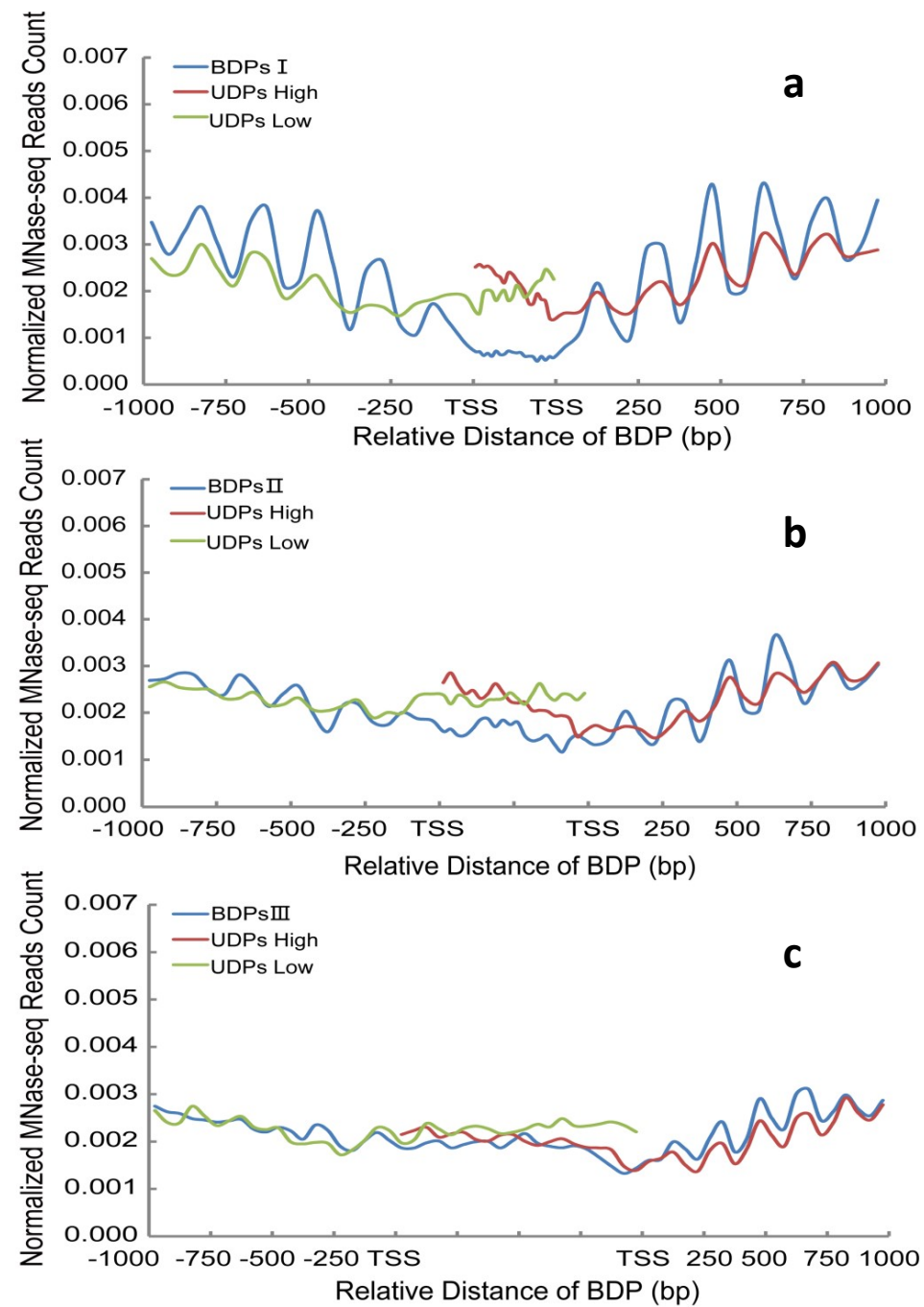

**Additional file 4: Figure S4.**

Supplement: Additional file 4: Table S4. — High frequency of overrepresented motifs within constitutive and tissue-specific BDPs. (PDF 334 kb) [file 12864_2016_3125_MOESM4_ESM.pdf]
