# Supplementary material for: Histone modifications facilitate the coexpression of bidirectional promoters in rice
Source: BMC Genomics. 2016 Sep 30;17:768. doi: 10.1186/s12864-016-3125-0 (PMC5045660; doi:10.1186/s12864-016-3125-0)
Supplement: Additional file 5: Table S5. — Overrepresented motifs in BDPs response to drought stress (PDF 348 kb) [file 12864_2016_3125_MOESM5_ESM.pdf]

Additional file 5: Table S1

Types of BDPs	Promoter regions (%)
**0~250(BDPs Ⅰ)**	
GC	54.22
TATA-box	17.59
**250~500(BDPs Ⅱ)**	
GC	49.77
TATA-box	51.84
**500~1000(BDPs Ⅲ)**	
GC	45.33
TATA-box	81.83
**Random Ⅰ**	
GC	47.05
TATA-box	46.50
**Random Ⅱ**	
GC	43.72
TATA-box	70.90
**Random Ⅲ**	
GC	42.03
TATA-box	90.50
