# Supplementary material for: Histone modifications facilitate the coexpression of bidirectional promoters in rice
Source: BMC Genomics. 2016 Sep 30;17:768. doi: 10.1186/s12864-016-3125-0 (PMC5045660; doi:10.1186/s12864-016-3125-0)
Supplement: Additional file 8: Table S7. — Fold difference of intensity of histone marks and nucleosome occupancy between bidirectional genes and unidirectional genes. (PDF 246 kb) [file 12864_2016_3125_MOESM8_ESM.pdf]

Additional file 8: Table S4:

Motif Name	Query count	SD	P-value	Motif seq
**Constitutive BDPs**				
**SORLIP2AT**	500	5.146584	1.00e-7	**GGGCC**
**SITEIIATCYTC**	47	10.58654	0	**TGGGCY**
**UP1ATMSD**	81	9.883992	0	**GGCCCAWWW**
**Seedling-specific BDPs**				
**TBF1HSF**	17	2.625993	4.32e-3	**GAAGAAGAA**
**Callus-specific BDPs**				
**ACGTABREMOTIFA2OSEM**	41	3.206062	6.73e-4	**ACGTGKC**
**BOXIIPCCHS**	31	3.055217	1.12e-3	**ACGTGGC**
