# Supplementary material for: Histone modifications facilitate the coexpression of bidirectional promoters in rice
Source: BMC Genomics. 2016 Sep 30;17:768. doi: 10.1186/s12864-016-3125-0 (PMC5045660; doi:10.1186/s12864-016-3125-0)
Supplement: Additional file 9: Table S8. — Comparison between ChIP-seq and ChIP-qPCR assay in an individual BDP locus. (PDF 247 kb) [file 12864_2016_3125_MOESM9_ESM.pdf]

Additional file 9: Table S5

Motif Name	Query count	Frequency	SD	p-value	Motif seq	regulated
SORLIP2AT	177/168	2.85/2.40	3.141766	8.40e-04	GGGCC	up/down
SITEIIATCYTC	151/150	2.44/2.14	5.037221	2.00e-07	TGGGCY	up/down
ABRERATCAL	94	1.52	2.226813	0.01298	MACGYGB	up
CACGTGMOTIF	46	0.74	1.886906	0.02959	CACGTG	up
ACGTABREMOTIFA2OSEM	42	0.68	4.183141	1.44e-05	ACGTGKC	up
CAMTA1	30	0.48	1.939313	0.02623	CCGCGT	up
BOXIIPCCHS	25	0.40	2.450225	0.00714	ACGTGGC	up
UP1ATMSD	22/22	0.35/0.31	3.893964	4.93e-05	GGCCCAWWW	up/down
IRO2OS	21	0.34	1.905147	0.02838	CACGTGG	up
GADOWNAT	17	0.27	4.552036	2.7e-06	ACGTGTC	up
ABREATCONSENSUS	15	0.24	2.47537	0.00665	YACGTGGC	up
EMBP1TAEM	10	0.16	2.231832	0.01281	CACGTGGC	up
HEXAT	10	0.16	2.161833	0.01532	TGACGTGG	up
UPRMOTIFIAT	10	0.16	2.161833	0.01532	CCACGTCA	up

Notes: up and down corresponding to up-regulation and down-regulation of gene expression under drought treatment as compared to the untreated control. Red-colored highlights represent overrepresented motifs in BDPs are not related to drought stress response
