# Supplementary material for: Histone modifications facilitate the coexpression of bidirectional promoters in rice
Source: BMC Genomics. 2016 Sep 30;17:768. doi: 10.1186/s12864-016-3125-0 (PMC5045660; doi:10.1186/s12864-016-3125-0)
Supplement: Additional file 10: Figure S2. — Profiling of histone marks across type II BDPs and UDP controls with the same gene number and same expression level as bidirectional gene pairs Unidirectional genes with higher and lower FPKM values were aligned on the right and left side, respectively (Additional file 10: Figure S2b, d and f). And bidirectional gene pairs with higher and lower FPKM values were aligned on the right and left sides of BDPs, respectively (Additional file 10: Figure S2a, c and e). Normalized reads counts indicated the enrichment of each mark were calculated by reads number per bp of genomic region per million reads. X-axes show the relative distance of BDPs (bp) in Additional file 10: Figure S2a, c and e and the position relative to TSS in Additional file 10: Figure S2b, d and f; Y-axes show normalized reads counts (read number in per bp genome in per million reads) within 1 kb upstream and downstream of TSS. A. Profiles of active marks: H4K12ac, H3K27ac, H3K4ac and H3K9ac in type II BDPs (Additional file 10: Figure S2a) and UDPs (Additional file 10: Figure S2b), respectively. B. Profiles of active marks: H3K4me2, H3K4me3 and H3K36me3 in type II BDPs (Additional file 10: Figure S2c) and UDPs (Additional file 10: Figure S2d), respectively. C. Profiles of repressive marks: H3K9me1, H3K9me3 and H3K27me3 in type II BDPs (Additional file 10: Figure S2e) and UDPs (Additional file 10: Figure S2f), respectively. (PDF 271 kb) [file 12864_2016_3125_MOESM10_ESM.pdf]

Additional file 10: Table S6

BDPS	Co-expression	Anti-expression	Independent expression	Null	Total
**BDPs-Ⅰ**	145 (50%)	20 (6.90%)	98 (33.79%)	27 (9.31%)	290
**BDPs-Ⅱ**	86(29.25%)	26(8.84%)	83(28.23%)	99 (33.67%)	294
**BDPs-Ⅲ**	133(21.21%)	49(7.81%)	188(29.98%)	257 (40.99%)	627
**Random**	148(14.80%)	121(12.1%)	341(34.10%)	390 (39.00%)	1000

Note: 1000 non-adjacent gene pairs were randomly selected for calculating Pearson correlation coefficient. Gene pairs with Pearson correlation coefficient greater than the average number of all positive values (0.38) were defined as co-expressed ones; gene pairs with Pearson correlation coefficient less than the average number of all negative values (-0.20) were defined as anti-expressed ones; gene pairs with Pearson correlation coefficient between -0.2 and 0.38 were defined as independent ones. Null indicate gene pairs cann't test the Pearson correlation coefficient.
