# Supplementary material for: Histone modifications facilitate the coexpression of bidirectional promoters in rice
Source: BMC Genomics. 2016 Sep 30;17:768. doi: 10.1186/s12864-016-3125-0 (PMC5045660; doi:10.1186/s12864-016-3125-0)
Supplement: Additional file 11: Figure S3. — Profiling of histone marks across type III BDPs and UDP controls with the same gene number and same expression level as bidirectional gene pairs Unidirectional genes with higher and lower FPKM values were aligned on the right and left side, respectively (Additional file 11: Figure S3b, d and f). Bidirectional gene pairs with higher and lower FPKM values were aligned on the right and left sides of BDPs, respectively (Additional file 11: Figure S3a, c and e). Normalized reads counts indicated the enrichment of each mark were calculated by reads number per bp of genomic region per million reads. X-axes show the relative distance of BDPs (bp) in Additional file 11: Figure S3a, a and e and the position relative to TSS in Additional file 11: Figure S3b, d and f; Y-axes show normalized reads counts (read number in per bp of genome per million reads) within 1 kb upstream and downstream of TSS. A. Profiles of active marks: H4K12ac, H3K27ac, H3K4ac and H3K9ac in type III BDPs (Additional file 11: Figure S3a) and UDPs (Additional file 11: Figure S3b), respectively. B. Profiles of active marks: H3K4me2, H3K4me3 and H3K36me3 in type III BDPs (Additional file 11: Figure S3c) and UDPs (Additional file 11: Figure S3d), respectively. C. Profiles of repressive marks: H3K9me1, H3K9me3 and H3K27me3 in type III BDPs (Additional file 11: Figure S3e) and UDPs (Additional file 11: Figure S3f), respectively. (PDF 313 kb) [file 12864_2016_3125_MOESM11_ESM.pdf]

Additional file 11: Table S7

Histone marks	250bp FPKM + (1×)	250bp FPKM + (5×)	250bp FPKM - (1×)	250bp FPKM - (5×)	500bp FPKM + (1×)	500bp FPKM + (5×)	500bp FPKM - (1×)	500bp FPKM - (5×)	1000bp FPKM + (1×)	1000bp FPKM + (5×)	1000bp FPKM - (1×)	1000bp FPKM - (5×)
H4K12ac	2.44	2.65	2.73	2.60	2.43	2.12	1.95	1.80	1.39	1.29	1.57	1.40
H3K27ac	2.42	2.28	2.11	2.03	1.49	1.42	1.45	1.36	1.14	1.10	1.28	1.20
H3K4ac	2.02	2.08	2.15	2.37	1.84	1.66	1.99	1.57	1.25	1.20	1.52	1.30
H3K9ac	1.98	1.95	2.11	2.27	1.68	2.01	1.75	1.47	1.17	1.15	1.50	1.28
H3K23ac	1.87	1.74	1.90	1.81	1.37	1.42	1.34	1.14	1.13	1.10	1.21	1.15
H4K16ac	1.86	1.72	1.84	1.77	1.28	1.32	1.28	1.16	1.13	1.08	1.14	1.11
H3K4me1	1.61	1.61	1.67	1.66	1.21	1.31	1.39	1.40	1.05	1.11	1.10	1.06
H3K4me2	1.55	1.59	1.50	1.42	1.34	1.47	1.31	1.15	1.08	1.07	0.94	0.97
H3K36me3	1.57	1.58	1.85	1.85	1.33	1.42	1.20	1.37	1.12	1.17	1.13	1.19
H3K4me3	1.41	1.41	1.44	1.50	1.06	1.17	1.35	1.37	1.00	1.07	1.13	1.20
H3K9me3	1.33	1.33	1.33	1.37	1.23	1.19	0.90	1.04	1.08	1.05	0.99	0.96
H3K9me1	1.19	1.31	1.22	1.26	0.93	1.13	1.04	1.20	1.09	1.06	1.10	1.07
H3K27me3	0.58	0.66	0.33	0.54	0.93	0.87	0.76	0.78	0.51	0.75	0.58	0.59
MNase-seq reads	1.33	1.45	1.27	1.44	1.18	1.44	1.07	1.07	1.06	1.17	1.00	1.02

Notes: "+" and "-" represent BDPs with high and low FPKM values, respectively.
