# Supplementary material for: Histone modifications facilitate the coexpression of bidirectional promoters in rice
Source: BMC Genomics. 2016 Sep 30;17:768. doi: 10.1186/s12864-016-3125-0 (PMC5045660; doi:10.1186/s12864-016-3125-0)
Supplement: Additional file 12: Table S9. — Kolmogorov-Smirnov test of eu-/hetero-chromatin marks and nucleosome occupancy reads between gene body of bidirectional gene pairs and control genes. (PDF 272 kb) [file 12864_2016_3125_MOESM12_ESM.pdf]

Additional file 12: Table S8

	ID of Gene pairs	H4K12ac		H3K27ac	
		ChIP-seq	ChIP-qPCR	ChIP-seq	ChIP-qPCR
		Normalized reads count	% Input	Normalized reads count	% Input
BDPs-1	LOC_Os10g36260(+)/LOC_Os10g36250(-)	0.047/0.021	10.44±4.09/5.58±0.96	0.008/0.03	21.34±4.35/58.39±19.16
BDPs-2	LOC_Os04g51270(+)/LOC_Os04g51280(-)	0.041/0.005	11.37±1.39/4.30±0.68	0.011/0.005	36.91±8.90/14.60±2.15
BDPs-3	LOC_Os08g03390(+)/LOC_Os08g03380(-)	0.054/0.036	12.33±0.39/9.94±1.58	0.014/0.01	42.53±1.30/32.28±4.59
BDPs-4	LOC_Os04g56646(+)/LOC_Os04g56640(-)	0.04/0.023	14.43±2.15/6.82±0.75	0.015/0.012	55.24±13.25/35.09±4.06
BDPs-5	LOC_Os03g58150(+)/LOC_Os03g58160(-)	0.036/0.017	22.96±5.43/14.02±1.18	0.007/0.001	82.88±20.15/42.44±9.90

Note: (+) represents the gene with higher FPKM
(-) represents the gene with lower FPKM
