# Supplementary material for: Histone modifications facilitate the coexpression of bidirectional promoters in rice
Source: BMC Genomics. 2016 Sep 30;17:768. doi: 10.1186/s12864-016-3125-0 (PMC5045660; doi:10.1186/s12864-016-3125-0)
Supplement: Additional file 13: Table S10. — Kolmogorov-Smirnov test of eu-/hetero-chromatin marks and nucleosome occupancy between gene body of coexpression and anti-expression bidirectional gene pairs. (PDF 325 kb) [file 12864_2016_3125_MOESM13_ESM.pdf]

Additional file 13: Table S9

Histone marks	250bp with high FPKM	250bp with low FPKM	500bp with high FPKM	500bp with low FPKM	1000bp with high FPKM	1000bp with low FPKM
**H4K12ac**	0.00186**	0.00006**	0.00949**	0.12010	0.61230	0.00205**
**H3K4ac**	0.00051**	0.00025**	0.09857	0.45210	0.70690	0.39360
**H4K16ac**	0.00456**	2.73e-05**	0.2083	0.5154	0.356	0.1405
**H3K4me2**	0.00036**	0.00252**	0.12010	0.00413**	0.75310	0.70690
**H3K36me3**	2.28e-06**	2.64e-09**	0.24680	0.00413**	0.22970	0.01997*
**Nucleosome occupancy**	1.48e-07**	0.00036**	0.71920	0.45210	0.61230	0.61230
**H3K27me3**	0.07275	5.35e-06**	0.58200	0.65060	0.61230	0.00560**
**H3K27ac**	0.56230	0.08978	0.45210	0.65060	0.75310	0.43360
**H3K9ac**	0.07275	0.02292	0.12010	0.65060	0.08160	0.39360
**H3K23ac**	0.11	0.4326	0.9696	0.4521	0.1405	0.09391
**H3K4me3**	0.11000	0.08978	0.33920	0.00024**	0.06102	0.07067
**H3K9me1**	0.04679*	0.08978	0.96960	0.00124**	0.14050	0.18080
**H3K9me3**	0.11000	0.07275	0.51540	0.02622*	0.35600	0.91020

Note: *$p<0.05$ representing significant change.
**$p<0.01$ representing extremely significant change.
