# Supplementary material for: Histone modifications facilitate the coexpression of bidirectional promoters in rice
Source: BMC Genomics. 2016 Sep 30;17:768. doi: 10.1186/s12864-016-3125-0 (PMC5045660; doi:10.1186/s12864-016-3125-0)
Supplement: Additional file 14: Figure S4. — Comparison of profiling of nucleosome positioning between BDPs and UDPs. Unidirectional gene controls or bidirectional gene pairs with higher and lower FPKM values were aligned on the right and left sides of BDPs, respectively. Normalized MNase-seq reads count representing nucleosome positioning were calculated by reads number per bp of genomic region per million reads. X-axes in Additional file 14: Figure S4a, b and c show relative distance of BDPs (bp); Y axes in Additional file 14: Figure S4a, b and c show normalized MNase-seq reads counts. The number and expression level of unidirectional genes analyzed were the same as the corresponding bidirectional gene pairs. A. Profile of MNase-seq reads around type I BDPs and corresponding UDPs control with higher or lower FPKM values, respectively. B. Profile of MNase-seq reads around type II BDPs and corresponding UDPs control with higher or lower FPKM values, respectively. C. Profile of MNase-seq reads around type III BDPs and corresponding UDPs control with higher or lower FPKM values, respectively. (PDF 324 kb) [file 12864_2016_3125_MOESM14_ESM.pdf]

Additional file 14: Table S10

Histone marks	Co-expression	Anti-expression
**H3K4ac**	0.31110	6.25e-08**
**H4K12ac**	0.70430	2.38e-06**
**H3K9ac**	0.57980	8.45e-06**
**H3K27ac**	0.31110	7.09e-05**
**H3K4me3**	0.64190	0.04474*
**H4K16ac**	0.3466	0.00022**
**H3K27me3**	0.35710	0.00029**
**H3K9me1**	0.16860	1.76e-05**
**H3K9me3**	0.23170	0.01226*
**H3K4me2**	0.00158**	0.00450**
**H3K36me3**	7.27e-05**	9.99e-16**
**H3K23ac**	2.75e-05**	0.03771*
**Nucleosome occupancy**	0.00767**	3.97e-06**

Note: *$p<0.05$ representing significant change.
**$p<0.01$ representing extremely significant change.
