# Supplementary material for: Histone modifications facilitate the coexpression of bidirectional promoters in rice
Source: BMC Genomics. 2016 Sep 30;17:768. doi: 10.1186/s12864-016-3125-0 (PMC5045660; doi:10.1186/s12864-016-3125-0)
Supplement: Additional file 15: Table S11. — Primer information used for the ChIP-qPCR assay. (PDF 246 kb) [file 12864_2016_3125_MOESM15_ESM.pdf]

Additional file 15: Table S11:

	Gene pairs ID	FPKM	Forward sequence	Reverse sequence	Amplificon (bp)
**BDPs-1**	LOC_Os10g36260(+)	147.01	AGAGTGGGAGCGGGACGT	AGGGAGGCGAGCTTGAGGG	148
	LOC_Os10g36250(-)	8.14	AACAAATCAACCTGCGAGTA	ATTGGTGAGCTGACCGAC	143
**BDPs-2**	LOC_Os04g51270(+)	139.91	CGACTAGGGAACAAGGAA	TGATAGTAGGCAGATTGAGTAA	136
	LOC_Os04g51280(-)	83.7	GGCCGGAGTCGTCGTACT	TCGATCATCTGCTGCTTGG	157
**BDPs-3**	LOC_Os08g03390(+)	102.67	GTGGATTTGGTGTAGATTTCG	AATTCAGGCAACGCAGAG	124
	LOC_Os08g03380(-)	22.03	CACCTCGTAGGGGTTCTCC	GGCATGGAATGAAGGGTC	123
**BDPs-4**	LOC_Os04g56646(+)	79.02	CGCGTTAGGGTTTCTTCG	TGCACCACCACCTTCTCC	153
	LOC_Os04g56640(-)	24.98	AGACTTGCCCAGGCTCAG	TTGAATCAATGCCCAGAT	146
**BDPs-5**	LOC_Os03g58150(+)	67.25	GAGGATGACGTAGATCTTGTG	GAAGAGGGAAGCCATGTT	130
	LOC_Os03g58160(-)	22.44	TCCTCGTCCTCAAATCTCC	GAACCCGACAGAATCAGAAA	131

Note: (+) represents the gene with higher FPKM
(-) represents the gene with lower FPKM
